# An audit of awareness about maternal sepsis in a UK district general hospital

**DOI:** 10.1186/cc10389

**Published:** 2011-10-27

**Authors:** C Donohue, E Teh, M Kitching

**Affiliations:** 1 Department of Anaesthesia, Lister Hospital, Stevenage, UK

## Introduction

The UK national body, which reviews maternal mortality (Centre for Maternal and Child Enquiries (CMACE)), has recently published their 2006 to 2008 report. This highlighted an increase in maternal sepsis, making it the leading cause of direct death amongst peripartum women in the UK (26 out of a total 107 direct deaths) [1]. The Surviving Sepsis Campaign (SSC) published updated sepsis resuscitation and management bundles in 2008 [2]. We decided to audit awareness about sepsis amongst staff caring for perpartum women.

## Methods

A questionnaire was devised and distributed to midwives, obstetricians and anaesthetists. This asked the criteria for the systemic inflammatory response syndrome (SIRS), common sites of maternal sepsis, the initial duties of care ('sepsis six' resuscitation bundle: delivery of oxygen, intravenous fluids, intravenous antibiotics, taking of blood cultures, measurement of plasma haemoglobin, lactate and urine output) and recognition and management of severe sepsis.

## Results

**Figure 1:**
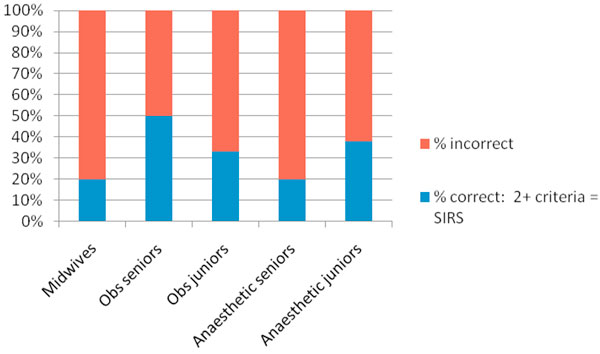


**Figure 2:**
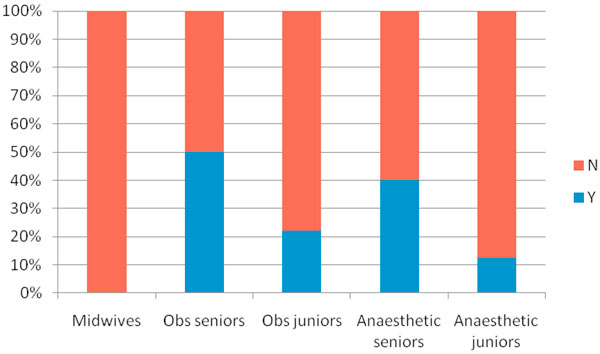


There was a 98% response rate with 41 completed questionnaires returned, 15 from midwives, 13 from obstetricians and 13 from anaesthetists. We found that awareness was suboptimal within all groups. Of the six criteria for SIRS, suggested by the SSC, two criteria (altered consciousness and hyperglycaemia) were poorly identified and few responders were aware that two or more criteria indicated SIRS (Figure 1). Most healthcare professionals correctly identified genital tract infection as the leading source of maternal sepsis. The majority of responders had not heard of the 'sepsis six' (Figure 2). Out of the initial duties of care, delivery of oxygen and monitoring urine output were poorly identified. Respondents were not confident in identifying features of severe sepsis and with the exception of hypotension despite fluids, other markers of end organ dysfunction were underreported.

## Conclusion

It is all of our responsibilities to focus efforts on the emerging threat of maternal sepsis as highlighted by CMACE. Historically, we have seen a significant improvement in maternal mortality rates when specific interventions have targeted those issues raised in previous CMACE reports (for example, venous thromboembolism). We therefore propose to develop local clinical guidelines, posters and factsheets with formal teaching sessions and multidisciplinary simulator workshops to raise awareness, optimise care and minimise preventable deaths from maternal sepsis. We will re-audit awareness in 6 months time to complete the audit cycle.
